# Association between alcohol consumption status and obesity-related comorbidities in men: data from the 2016 Korean community health survey

**DOI:** 10.1186/s12889-021-10776-y

**Published:** 2021-04-15

**Authors:** Bo-Yeon Kim, Hyewon Nam, Jeong-Ju Yoo, Yoon-Young Cho, Dug-Hyun Choi, Chan-Hee Jung, Ji-Oh Mok, Chul-Hee Kim

**Affiliations:** 1 Division of Endocrinology and Metabolism, Department of Internal Medicine, Soonchunhyang University Bucheon Hospital, Soonchunhyang University College of Medicine, 170 Jomaru-ro, Wonmi-gu, Bucheon, 14584 Republic of Korea; 2 Data Science Team, Hanmi Pharm. Co., Ltd., Seoul, Republic of Korea; 3 Division of Gastroenterology and Hepatology, Department of Internal Medicine, Soonchunhyang University Bucheon Hospital, Soonchunhyang University College of Medicine, Bucheon, Republic of Korea

**Keywords:** Alcohol drinking, Diabetes mellitus, Dyslipidemias, Hypertension, Obesity, Surveys and questionnaires

## Abstract

**Background:**

This study was performed to investigate the association between the amount of alcohol consumption or binge drinking and obesity-related comorbidities in Korean men.

**Methods:**

A total of 103,048 men aged 19 years or older were investigated in the 2016 Korean Community Health Survey. The participants were divided into five groups according to the standard number of alcoholic drinks consumed per week.

**Results:**

Of the total participants, 20.7% were in the high alcohol consumption group, consuming more than 28 drinks per week. After adjustment for clinical factors, high alcohol consumption was significantly associated with higher odds ratios (ORs) of obesity (OR, 1.449; 95% confidence interval [CI], 1.412 to 1.591; *P* < 0.0001), hypertension (OR, 1.76; 95% CI, 1.636 to 1.894; *P* < 0.0001), and dyslipidemia (OR, 1.356; 95% CI, 1.247 to 1.474; *P* < 0.0001). In contrast, mild to moderate alcohol consumption was associated with a lower risk of diabetes (OR, 0.799; 95% CI, 0.726 to 0.88; *P* = 0.0015) and high alcohol consumption was not associated with a higher risk of diabetes (OR, 0.945; 95% CI, 0.86 to 1.039; *P* = 0.0662). Among drinkers, except for social drinkers, binge drinking was significantly associated with higher risks of obesity, hypertension, diabetes, and dyslipidemia.

**Conclusions:**

High alcohol consumption was associated with higher risks of obesity, hypertension, and dyslipidemia in Korean men. In contrast, high consumption was not associated with a higher risk of diabetes. In particular, binge drinkers were associated with higher risks of obesity, hypertension, diabetes, and dyslipidemia compared to non-binge drinkers.

## Background

The prevalence of alcohol dependence and alcohol abuse is reported to be much higher in Korea compared to other countries [1]. Until recent years, social awareness of drinking has been low despite the incidence of alcohol-related accidents [2]. Recently, alcohol consumption in Organization for Economic Co-operation and Development (OECD) countries has been decreasing overall, but alcohol consumption in Korea is increasing [3]. In addition, the proportion of high-risk drinkers is high even in diabetic patients, especially in men (men 24.3% and women 3.1%) according to the Diabetes Fact Sheets in Korea, 2018 [4].

Several studies have shown an association between alcohol consumption and obesity-related comorbidities, such as diabetes, hypertension, dyslipidemia, and cardiovascular diseases. However, there is still controversy about these associations. There is also controversy about the effect of alcohol consumption on obesity [5, 6]. The discrepancy among previous studies may be due to the differences in drinking patterns [7, 8], behaviors and the culture of drinking, which varies in each country. In Korea, the proportion of binge drinkers and heavy drinkers is reported to be particularly high [3]. In a previous study, drinking frequency was inversely associated with obesity, whereas the amount of alcohol intake was positively associated with obesity [9]. However, there are a few studies on association between alcohol consumption pattern, in particular binge drinking, and obesity-related comorbidities.

This study aimed to 1) examine the association between the amount of alcohol consumption and obesity-related comorbidities and 2) clarify the association between binge drinking and obesity-related comorbidities, in Korean men by analyzing nationally representative data (Korea Community Health Survey [KCHS] 2016).

## Methods

### Korean community health survey

The KCHS is a nationwide survey for health and disease statistics conducted since 2008 by the Korean Centers for Disease Control and Prevention (KCDC) to understand the current state of chronic diseases and to establish community healthcare plans [10]. To standardize community surveys, KCDC supported conducting the surveys, the item guidelines, interviewer training material, and the data analysis syntax. By multistage sampling design, small villages (Tong Ban/Lee, primary sampling unit) and households in the area (final sampling unit) were randomly selected in 16 metropolitan cities and provinces with 254 regional sites. The KCHS standardized questionnaire made by the KCDC staff was used to assess the prevalence of personal health practices and behaviors, including smoking, alcohol consumption, driving, blood pressure, physical activity, weight control, quality of life (QoL), medical services, accidents, and injuries. The KCHS was conducted by trained interviewers who visited the selected sample households and carried out one-on-one interviews with all adults aged ≥19 years using an indirect entry method. The interviewer measured the height and weight at each visit. The interview took approximately 20 to 30 min per person [10].

### Subjects

This study was conducted on people who participated in the 2016 KCHS. A total of 228,452 individuals (103,048 men and 125,404 women) aged 19 years or older were investigated in the 2016 KCHS. Among them, 103,048 men were included and analyzed in this study. This study was approved by the Institutional Review Board (IRB) of Soonchunhyang University Bucheon Hospital (IRB No. 2020–01–016-001). The requirement for informed consent was waived because the data in this database were de-identified.

### Study variables

The amount of alcohol consumption (number of drinks/day, and frequency of drinking), the presence of hypertension, diabetes, dyslipidemia, and obesity were analyzed from the KCHS. The current smoker was defined as those who have smoked five packs (100 cigarettes) or more in their lifetime and currently smoke every day or on occasion. According to the 2019 Practice Guidance from the American Association for the Study of Liver Diseases (AASLD guideline) and the European Association for the Study of the Liver Clinical Practice Guidelines (EASL guideline), one standard drink was defined as about 7–10 g alcohol [11, 12]. The monthly drinking rate was defined as the percentage of participants who drink at least once a month. Alcohol consumption was measured using the following parameters: a history of drinking, binge drinking, number of drinks/day, and frequency of drinking. Binge drinking was defined when the average amount of alcohol consumed on one occasion was more than seven drinks in men. High-risk drinking in this study was defined when the average amount of alcohol consumed was more than seven drinks on each of two or more occasions per week. In KCHS, the presence of hypertension, diabetes, and dyslipidemia were defined as being diagnosed by a physician or having a history of treatment. Obesity was defined as a body mass index (BMI) of 25 kg/m^2^ or greater based on the World Health Organization (WHO) Asia-Pacific criteria for obesity [13] and the Korean Society for the Study of Obesity guideline 2018 [14]. Education level (no formal education, elementary school, middle school, high school, college, and above college) and monthly household income (low < 1850, low-middle 1850 to 3699, middle-high 3700 to 5549, and high ≥5550 US dollars) were assessed. Regular exercise was defined as participating in moderate physical activity for ≥5 days per week and ≥ 30 min per activity or in vigorous activity for ≥3 days per week and ≥ 20 min per activity. Psychiatric problems were assessed by depressive symptoms, subjective stress level, suicidal ideation, and clinic visit for psychiatric illness.

### Statistical analysis

SAS Enterprise Guide 7.1 (SAS Inc., Cary, NC, USA) was used for all statistical analyses. The analyses used sampling weights to account for the complex sampling design of the KCHS. The level of significance was set to 0.05. We analyzed using multiple imputation of missing data (3127 individuals, 3.03%) with (the presence of alcohol consumption, frequency of drinking, the amount of alcohol consumption, weight, height, age, exercise, hypertension, diabetes, dyslipidemia, cardiovascular disease, current smoking status, education level, monthly familial income level, and psychiatric problem) to create 5 imputed data sets. We assessed the association of alcohol consumption with categorical variables using chi-squared tests. One-way analysis of variance was used to test the differences of means among groups according to the alcohol consumption levels. Multiple binary logistic regression analyses were used to identify the associations of alcohol consumption with obesity, hypertension, diabetes, and dyslipidemia with adjustment for covariates.

## Results

The baseline characteristics of the study participants are presented in Table 1. The 103,048 men aged 19 years or older were analyzed and divided into five groups according to the standard number of alcoholic drinks consumed per week. The proportion of participants in the non-drinker group was 20.7% and the proportion in the high alcohol consumption group, with more than 28 drinks per week, was 15.4% (Table 1). The percentage of smoking increased as alcohol consumption increased in men. The proportion of currently smoking individuals was the lowest in the non-drinker group. The non-drinker group had lower monthly household income levels and lower education levels than the other groups (Table 1). In men, the percentages of high-risk drinking (the average amount of alcohol consumed was ≥7 drinks and ≥ 2 times per week) and binge drinking (the average amount of alcohol consumed was ≥7 drinks in a single session) were 22.3 and 36.8%, respectively.

**Table 1:** Characteristics according to alcohol consumption status

	Alcohol consumption, standard drinks per week						***P*** value
	Total	None	0 < to 6	7 to < 14	14 to < 28	≥28	
*n* (%)	103,048 (100)	21,331 (20.7)	41,781 (46.4)	8908 (8.6)	15,147 (14.7)	15,881 (15.4)	
Age, yr	46.2 ± 0.1	56.1 ± 0.2	44.1 ± 0.1	41.0 ± 0.2	47.6 ± 0.1	44.6 ± 0.1	< 0.0001
BMI, kg/m^2^ (%)	24.11 ± 0.01	23.67 ± 0.03	23.98 ± 0.02	24.57 ± 0.03	24.08 ± 0.03	24.59 ± 0.03	< 0.0001
≥ 25	33.05	27.73	32.08	38.43	32.61	40.17	
< 25	66.95	72.27	67.92	61.57	67.39	59.83	
Smoking (%)							< 0.0001
Current smoker	37.20	23.37	32.04	43.29	47.39	56.20	
Ex-smoker	37.87	47.04	36.37	33.19	37.80	32.21	
Never smoker	24.93	29.59	31.59	23.52	14.81	11.59	
Exercise (%)							< 0.0001
None	60.62	68.23	58.65	57.18	58.12	59.90	
< 3 times per wk	12.48	7.74	13.93	14.44	13.87	12.64	
≥ 3 times per wk	26.89	24.03	27.42	28.38	28.01	27.46	
Education (%)							< 0.0001
≤ High school	65.41	79.23	59.90	58.49	64.56	66.04	
≥ University	30.63	16.90	35.29	37.76	32.02	31.47	
≥ Graduate school	3.96	3.87	4.81	3.75	3.42	2.49	
Monthly household income^a^ (US dollars) (%)							< 0.0001
Low	33.19	54.69	29.43	23.89	28.07	24.32	
Low-middle	36.90	29.02	37.61	40.42	39.66	40.99	
Middle-high	19.01	10.31	20.71	23.18	20.78	22.19	
High	10.90	5.98	12.25	12.51	11.49	12.50	
Psychiatric problem (%)	1.62	2.10	1.57	1.48	1.22	1.59	< 0.0001
Obesity^f^ (%)	33.05	27.73	32.08	38.43	32.61	40.17	< 0.0001
Hypertension (%)	25.58	34.00	21.72	20.77	26.98	25.79	< 0.0001
Diabetes (%)	11.29	17.89	9.69	8.46	9.53	9.89	< 0.0001
Dyslipidemia (%)	14.14	15.36	13.36	13.67	14.18	14.75	< 0.0001

Values are presented as mean ± standard deviation or %

BMI, body mass index; ^f^Body mass index ≥25

^a^Monthly household income (US dollars): low < 1850, low-middle 1850 to 3699, middle-high 3700 to 5549, high ≥5550

After adjustment for clinical factors, such as age, smoking, exercise, psychiatric problems, education level, income level, and BMI, the high alcohol consumption group (alcohol consumption ≥28 glasses per week) was significantly associated with higher odds ratios (ORs) of hypertension (OR, 1.76; 95% confidence interval [CI], 1.636 to 1.894; *P* < 0.0001), and dyslipidemia (OR, 1.356; 95% CI, 1.247 to 1.474; *P* < 0.0001). After adjustment for clinical factors, such as age, smoking, exercise, psychiatric problems, education level, and income level, the high alcohol consumption group was significantly associated with higher risks of obesity (OR, 1.499; 95% CI, 1.412 to 1.591; *P* < 0.0001). However, the social drinker group (alcohol consumption 0 to 6 standard drinks per week) was not associated with a higher risk of obesity (OR, 1.01; 95% CI, 0.96 to 1.062; *P* = 0.6710). In contrast, after adjustment for clinical factors, the mild to moderate alcohol consumption group (alcohol consumption 0 to < 28 standard drinks per week) was associated with a lower risk of diabetes (OR, 0.799; 95% CI, 0.726 to 0.88; *P* = 0.0015) and only the high consumption group (≥28) was not associated with a risk of diabetes (OR, 0.945; 95% CI, 0.86 to 1.039; *P* = 0.0662) (Table 2).

**Table 2:** Odds ratios (95% confidence interval) for obesity, hypertension, diabetes, and dyslipidemia according to alcohol consumption status

Drinks/wk	Model 1^**a**^	Model 2^**b**^	Model 3^**c**^	Model 4^**d**^	Model 5^**e**^
	OR (95% CI)	OR (95% CI)	OR (95% CI)	OR (95% CI)	OR (95% CI)
None	1.00 (reference)	1.00 (reference)	1.00 (reference)	1.00 (reference)	
0 > to 6	1.071 (1.019–1.126)	1.063 (1.011–1.117)	1.061 (1.009–1.116)	1.01 (0.96–1.062)	
7 to < 14	1.404 (1.312–1.503)	1.387 (1.295–1.485)	1.386 (1.294–1.484)	1.326 (1.238–1.421)	
14 to < 28	1.161 (1.094–1.233)	1.149 (1.082–1.221)	1.147 (1.079–1.219)	1.088 (1.023–1.157)	
≥28	1.56 (1.473–1.653)	1.557 (1.468–1.652)	1.556 (1.467–1.651)	1.499 (1.412–1.591)	
None	1.00 (reference)	1.00 (reference)	1.00 (reference)	1.00 (reference)	1.00 (reference)
0 > to 6	1.066 (1.005–1.13)	1.064 (1.004–1.128)	1.072 (1.011–1.137)	1.082 (1.02–1.147)	1.079 (1.016–1.146)
7 to < 14	1.36 (1.246–1.484)	1.372 (1.257–1.498)	1.385 (1.268–1.512)	1.394 (1.277–1.523)	1.283 (1.173–1.403)
14 to < 28	1.487 (1.388–1.593)	1.522 (1.419–1.632)	1.539 (1.435–1.651)	1.55 (1.444–1.663)	1.536 (1.43–1.65)
≥28	1.823 (1.701–1.955)	1.897 (1.767–2.063)	1.914 (1.782–2.055)	1.919 (1.787–2.061)	1.76 (1.636–1.894)
None	1.00 (reference)	1.00 (reference)	1.00 (reference)	1.00 (reference)	1.00 (reference)
0 > to 6	0.837 (0.778–0.9)	0.825 (0.767–0.888)	0.843 (0.783–0.907)	0.854 (0.793–0.919)	0.848 (0.788–0.913)
7 to < 14	0.935 (0.833–1.049)	0.899 (0.801–1.009)	0.919 (0.819–1.032)	0.931 (0.828–1.045)	0.874 (0.777–0.983)
14 to < 28	0.834 (0.76–0.915)	0.787 (0.716–0.865)	0.807 (0.734–0.888)	0.818 (0.743–0.9)	0.799 (0.726–0.88)
≥28	1.058 (0.966–1.159)	0.987 (0.9–1.084)	1.007 (0.917–1.105)	1.015 (0.924–1.116)	0.945 (0.86–1.039)
None	1.00 (reference)	1.00 (reference)	1.00 (reference)	1.00 (reference)	1.00 (reference)
0 > to 6	1.245 (1.164–1.331)	1.229 (1.149–1.315)	1.233 (1.152–1.319)	1.164 (1.088–1.246)	1.165 (1.087–1.248)
7 to < 14	1.428 (1.294–1.575)	1.39 (1.26–1.534)	1.397 (1.266–1.543)	1.327 (1.201–1.465)	1.24 (1.121–1.371)
14 to < 28	1.354 (1.249–1.468)	1.313 (1.21–1.425)	1.32 (1.216–1.433)	1.242 (1.143–1.349)	1.222 (1.125–1.328)
≥28	1.577 (1.457–1.707)	1.534 (1.414–1.663)	1.544 (1.423–1.675)	1.474 (1.357–1.6)	1.356 (1.247–1.474)

^a^Model 1: adjusted by age

^b^Model 2: adjusted by age, smoking

^c^Model 3: adjusted by age, smoking, exercise, and psychiatric problem

^d^Model 4: adjusted by age, smoking, exercise, psychiatric problem, education level, and income level

^e^Model 5: adjusted by age, smoking, exercise, psychiatric problem, education level, income level, and BMI

^f^Body mass index ≥25

Of all the participants, the percentage of participants consuming more than seven drinks per week was 38.8% (*n* = 39,936). We analyzed the risk of binge drinking only for participants who drank more than seven drinks a week, excepting non-drinkers and social drinkers. Among them, 77.7% (*n* = 31,028) were binge drinkers. After adjusting for clinical factors, such as age, smoking, exercise, psychiatric problems, education level, income level, and BMI, binge drinking was significantly associated with higher risks of hypertension, diabetes, and dyslipidemia. After adjusting for clinical factors, such as age, smoking, exercise, psychiatric problems, education level, and income level, binge drinking was significantly associated with higher risk of obesity (Table 3). The adjusted ORs for obesity, hypertension, diabetes, and dyslipidemia were 1.424 (95% CI, 1.315 to 153), 1.133 (1.01 to 1.201), 1.189 (1.11 to 1.342) and 1.199 (1.089 to 1.294), respectively (Table 3).

**Table 3:** Odds ratios (95% confidence interval) of obesity, hypertension, diabetes, and dyslipidemia according to the presence of binge drinking among participants consuming more than 7 drinks per week (*n* = 39,936)

Binge drinking^**a**^	Model 1^**b**^		Model 2^**c**^		Model 3^**d**^		Model 4^**e**^		Model 5^**f**^	
	OR (95% CI)	***P*** value	OR (95% CI)	***P*** value	OR (95% CI)	***P*** value	OR (95% CI)	***P*** value	OR (95% CI)	***P*** value
**Obesity**^**g**^										
No (*n* = 8908)	1.00 (reference)		1.00 (reference)		1.00 (reference)		1.00 (reference)			
Yes (*n* = 31,028)	1.375 (1.28–1.507)	< 0.0001	1.420 (1.426–1.609)	< 0.0001	1.421 (1.31–1.508)	< 0.0001	1.424 (1.315–1.538)	< 0.0001		
**Hypertension**										
No (*n* = 8908)	1.00 (reference)		1.00 (reference)		1.00 (reference)		1.00 (reference)		1.00 (reference)	
Yes (*n* = 31,028)	1.991 (1.144–1.285)	< 0.0001	1.207 (1.17–1.321)	< 0.0001	1.226 (1.145–1.301)	< 0.0001	1.227 (1.146–1.311)	< 0.0001	1.133 (1.01–1.201)	< 0.0001
**Diabetes**										
No (*n* = 8908)	1.00 (reference)		1.00 (reference)		1.00 (reference)		1.00 (reference)		1.00 (reference)	
Yes (*n* = 31,028)	1.272 (1.139–1.462)	< 0.0001	1.274 (1.146–1.501)	< 0.0001	1.267 (1.141–1.499)	< 0.0001	1.256 (1.115–1.421)	< 0.0001	1.189 (1.11–1.342)	< 0.0001
**Dyslipidemia**										
No (*n* = 8908)	1.00 (reference)		1.00 (reference)		1.00 (reference)		1.00 (reference)		1.00 (reference)	
Yes (*n* = 31,028)	1.222 (1.11–1.301)	< 0.0001	1.183 (1.147–1.301)	< 0.0001	1.179 (1.117–1.299)	< 0.0001	1.181 (1.117–1.309)	< 0.0001	1.199 (1.089–1.294)	< 0.0001

^a^The definition of a binge drinking is that the average amount of alcohol consumed per session is more than seven drinks in men. OR, odds ratio; CI, confidence interval; BMI, body mass index

^b^Model 1: adjusted by age

^c^Model 2: adjusted by age, smoking

^d^Model 3: adjusted by age, smoking, exercise, and psychiatric problem

^e^Model 4: adjusted by age, smoking, exercise, psychiatric problem, education level, and income level

^f^Model 5: adjusted by age, smoking, exercise, psychiatric problem, education level, income level, and BMI

^g^Body mass index ≥25

## Discussion

The present study assessed the association between the amount of alcohol consumption and obesity-related comorbidities in Korean men. We also clarified the association between binge drinking and obesity-related comorbidities. In this population of Korean men, the proportion of the non-drinker group was 20.7% and the proportion of the high alcohol consumption group, with more than 28 drinks per week, was 15.4%. The percentage of high-risk drinkers (the average amount of alcohol consumed was ≥7 drinks on an occasion and ≥ 2 times per week) in men was 22.3% in this study. In the 2012 Korea National Health and Nutrition Examination Survey, the high-risk drinking rate was 21.8% for men and 6.0% for women [15], similar to our results. Compared to the average high-risk drinking rate in men across the world according to the WHO definition (16.1% for men), the high-risk drinking rate of Korean men was somewhat higher [3]. However, in defining the risk level of drinking, the WHO criteria were based on grams of pure alcohol (60 g of alcohol once a week or more) consumed and alcohol contained in a standard drink (one drink contains ~ 14 g of pure alcohol) according to the US National Institute on Alcohol Abuse and Alcoholism (NIAAA). Since there is no unified standard in Korea, the prevalence of high-risk drinking could vary depending on the definition of the investigators. Therefore, it is urgently necessary to form a consensus in Korea and perform further studies using the standardized definition.

It is well known that obesity is caused by a variety of factors, and in particular, personal habits such as smoking and exercise and a variety of psychosocial factors have a large effect on obesity [16]. In this study, personal habits such as smoking and exercise and psychosocial factors were assessed. In our study, the proportion of smokers increased as the amount of alcohol consumption increased. Alcohol drinking and smoking are highly correlated behaviors [17]. Several mechanisms may contribute to the link between alcohol drinking and smoking. These mechanisms include genes that regulate certain brain systems, conditioning mechanisms of cravings for alcohol or nicotine, and psychosocial factors, such as personality disorders [18]. In our results, another interesting finding was that the non-drinker group had lower monthly household income levels and lower education levels than the other groups. A previous systematic review reported that male frequent alcohol drinkers earned more money than non-drinkers [19]. These results suggest that social drinking may be associated with social capital and income.

Our major finding was that high alcohol consumption was significantly associated with higher risks of obesity, hypertension, and dyslipidemia in Korean men after adjustment for clinical factors. In contrast, high alcohol consumption was not associated with a higher risk of diabetes. On the other hand, binge drinking was significantly associated with higher risks of obesity, hypertension, diabetes, and dyslipidemia compared to non-binge drinkers, after adjustment for clinical factors.

The association between alcohol consumption status and obesity has been studied thoroughly in various populations. Among several cross-sectional studies, a common finding was that alcohol intake was not associated with BMI in men [7, 8, 20]. In contrast, some studies showed a positive correlation between alcohol intake and obesity in men, similar to our study [21]. Differences in alcohol intake patterns might be responsible for the discrepancy in the findings. Some researchers suggest that light, frequent alcohol drinking had a protective effect on obesity [22, 23]. In our study, the social drinker group was not associated with a higher risk of obesity. In contrast, heavy drinking and binge drinking were associated with a higher risk of obesity in this study. These findings are consistent with recent reports [23, 24]. Our results showed that a high total alcohol intake was associated with the risk of obesity and obesity-related comorbidities, and in particular, the amount of alcohol consumed in a single session was also important. Several hypotheses can explain the mechanisms by which high alcohol intake promotes weight gain. First, energy from alcohol can influence weight gain [25]. Second, as well as adding energy, alcohol may stimulate food intake [25]. Third, alcohol inhibits fat oxidation and affects energy storage [26]. Finally, binge drinking may lead to binge eating [27] and alcohol-induced overeating was shown to be related to the Agouti-related protein-cell activity in recent research [28].

Alcohol has effects on several lipid metabolism pathways in addition to elevating triglycerides [29]. Recent studies reported that heavy alcohol drinking had detrimental effects on lipid-related indices in men [30, 31]. Our results are consistent with those findings. The association between high alcohol consumption and hypertension has been established in epidemiological and clinical studies [32]. In particular, repeated binge drinking in men was associated with high blood pressure in a recent epidemiologic study [31]. Our study also confirmed that high alcohol consumption and binge drinking were significantly associated with a higher risk of hypertension after adjustment for clinical factors. There are several mechanisms by which alcohol can raise blood pressure, such as by an imbalance in the central nervous system, impaired baroreceptors, enhanced sympathetic activity, inflammation and oxidative stress of the vascular endothelium, enhanced renin-angiotensin-aldosterone system, and increased cortisol levels [32].

Notably, our results showed that the lower to moderate alcohol consumption group (alcohol consumption 0 to < 28 standard drinks per week) was associated with a lower risk of diabetes. Moreover, even in the high alcohol consumption group, there was no increased risk of diabetes. However, the binge drinking group was associated with a higher risk of diabetes compared to non-binge drinking group in this study. In a systematic review, moderate alcohol consumption protected against type 2 diabetes [33] and moderate amounts of alcohol may improve insulin sensitivity in previous studies [34, 35]. In some studies, heavy alcohol drinking increased the risk for type 2 diabetes and truncal obesity. Similarly, in our study, although the risk of diabetes was not increased in the high alcohol consumption group, the protective effect on diabetes found in the low to moderate alcohol consumption group was not seen in this group. The effect of alcohol drinking on diabetes risk seems to show a J- or U-shaped curve and binge drinking adversely affects risk of diabetes.

Our study had several limitations that need to be considered. First, due to its cross-sectional design, a causal relationship between alcohol consumption status and obesity-related comorbidities could not be determined. Second, there is no unified standard in Korea to define the risk levels of alcohol consumption. Therefore, the results should be interpreted with caution and further studies are needed. Third, the data on health-related behaviors and the diagnosis of obesity-related diseases were collected by surveys, not medical records. Nonetheless, because the KCHS was conducted as one-on-one interviews by trained personnel, the information on alcohol consumption, psychosocial factors, and anthropometrics could be highly reliable.

## Conclusions

In summary, high alcohol consumption was significantly associated with higher risks of obesity, hypertension, and dyslipidemia in Korean men. However, high alcohol consumption was not associated with a higher risk of diabetes and mild to moderate alcohol consumption was associated with a lower risk of diabetes. In particular, binge-drinkers were significantly associated with higher risks of obesity, hypertension, diabetes, and dyslipidemia compared to non-binge drinkers. In conclusion, our results showed that heavy alcohol use and binge drinking were associated with adverse effects on obesity-related comorbidities in Korean men. Follow-up studies are necessary to examine the long-term effects of alcohol consumption on obesity-related diseases and the glycemic control of diabetic patients in Korea.

## Data Availability

The data that support the findings of this study are available from the Korean Centers for Disease Control and Prevention (KCDC) but restrictions apply to the availability of these data, which were used under license for the current study, and so are not publicly available. Permissions from the KCDC are required to access the data (http://chs.cdc.go.kr).

## Abbreviations

OECD: Organization for Economic Co-operation and Development
KCHS: Korea Community Health Survey
KCDC: Korean Centers for Disease Control and Prevention
QoL: Quality of life
IRB: Institutional Review Board
BMI: Body mass index
WHO: World Health Organization
OR: Odds ratio
CI: Confidence interval
NIAAA: National Institute on Alcohol Abuse and Alcoholism
